# The Effect of *Helicobacter pylori* Eradication on the Levels of Essential Trace Elements

**DOI:** 10.1155/2014/513725

**Published:** 2014-06-26

**Authors:** Meng-Chieh Wu, Chun-Yi Huang, Fu-Chen Kuo, Wen-Hung Hsu, Sophie S. W. Wang, Hsiang-Yao Shih, Chung-Jung Liu, Yen-Hsu Chen, Deng-Chyang Wu, Yeou-Lih Huang, Chien-Yu Lu

**Affiliations:** ^1^Department of Internal Medicine, Kaohsiung Municipal Hsiao-Kang Hospital, Kaohsiung 812, Taiwan; ^2^Division of Gastroenterology, Department of Internal Medicine, Kaohsiung Medical University Hospital, Kaohsiung 807, Taiwan; ^3^Department of Medical Laboratory Science and Biotechnology, Kaohsiung Medical University, Kaohsiung 807, Taiwan; ^4^School of Medicine, College of Medicine, E-Da Hospital, I-Shou University, Kaohsiung 824, Taiwan; ^5^Graduate Institute of Public Health, Kaohsiung Medical University, Kaohsiung 807, Taiwan; ^6^Department of Medicine, Faculty of Medicine, College of Medicine, Kaohsiung Medical University, Kaohsiung 807, Taiwan; ^7^Division of Infectious Diseases, Department of Internal Medicine, Kaohsiung Medical University Hospital, Kaohsiung 807, Taiwan

## Abstract

*Objective*. This study was designed to compare the effect of *Helicobacter pylori* (*H. pylori*) infection treatment on serum zinc, copper, and selenium levels. *Patients and Methods*. We measured the serum zinc, copper, and selenium levels in *H. pylori*-positive and *H. pylori*-negative patients. We also evaluated the serum levels of these trace elements after *H. pylori* eradication. These serum copper, zinc, and selenium levels were determined by inductively coupled plasma mass spectrometry. *Results*. Sixty-three *H. pylori*-positive patients and thirty *H. pylori*-negative patients were studied. Serum copper, zinc, and selenium levels had no significant difference between *H. pylori*-positive and *H. pylori*-negative groups. There were 49 patients with successful *H. pylori* eradication. The serum selenium levels were lower after successful *H. pylori* eradication, but not significantly (*P* = 0.06). There were 14 patients with failed *H. pylori* eradication. In this failed group, the serum selenium level after *H. pylori* eradication therapy was significantly lower than that before *H. pylori* eradication therapy (*P* < 0.05). The serum zinc and copper levels had no significant difference between before and after *H. pylori* eradication therapies. *Conclusion*. *H pylori* eradication regimen appears to influence the serum selenium concentration (IRB number: KMUH-IRB-20120327).

## 1. Introduction 

*Helicobacter pylori (H. pylori)*, a spiral-shaped pathogenic bacterium found on the human gastric mucosa, was first isolated by Warren and Marshall in 1982. It is one of the most common worldwide human infections [1].* H. pylori* plays an important role in the development of chronic gastritis, gastric ulcer, duodenal ulcer, gastric adenocarcinoma, and gastric mucosa-associated lymphoid tissue lymphoma. Established indications for* H. pylori* eradication include gastric ulcer, duodenal ulcer, gastric mucosa-associated lymphoid tissue lymphoma, atrophic gastritis, after gastric cancer resection, uninvestigated dyspepsia, patients who are first degree relatives of patients with gastric cancer, and patient's wishes [2, 3]. Therefore, there are many studies concerning the treatment of* H. pylori* infection.

Copper is an essential nutrient in human physiology and is an essential constituent of numerous enzymes. Copper deficiency may result in microcytic anemia, leukopenia, osteoporosis, new subperiosteal bone formation, and fibrosis of epiphysis [4]. Zinc is also an important micronutrient in the human body and plays a vital role in homeostasis, immune function, oxidative stress, apoptosis, and aging. Zinc deficiency is related to atherosclerosis, several malignancies, neurological disorders, autoimmune diseases, aging, age-related degenerative diseases, and Wilson's disease [5]. Zinc is also a key component of proteins in adult humans [6]. Selenium is an important antioxidant and anticancer nutrient [7]. In the study of Steevens et al. selenium status has an inverse association with gastric and esophageal cancer [8].

*Helicobacter pylori* infection leads to chronic inflammation of gastric mucosa and peptic ulcer disease. It may influence the absorption of essential trace elements. The association between trace elements and* H. pylori* infection has been reported [9]. The aim of this study is to explore the effect of* H. pylori* eradication on the essential trace elements of zinc, copper, and selenium.

## 2. Method

### 2.1. Patients and Study Design

Patients, who were older than 18 years and complained of dyspepsia or epigastralgia, were evaluated in our gastroenterology outpatient clinic. The exclusion criteria included (a) age less than 18 years; (b) patients who had had previous* H. pylori* eradication; (c) patients with allergy history to PPI, or antibiotics; (d) patients who had ever taken PPI, antibiotics, and bismuth in the recent 4 weeks; (e) patients with severe systemic diseases (e.g., decompensated liver cirrhosis and uremia); (f) patients with previous gastric surgery; and (g) pregnant women. All of the clinical diagnoses were documented by endoscopic examination. The patients with* H. pylori* infection were classified as the study group, and the patients without* H. pylori* infection were the control group.

The status of* H. pylori* infection was diagnosed by histology, culture, and rapid urease test. Patients who were infected with* H. pylori* were treated with lansoprazole 30 mg bid, amoxicillin 1 g bid, and clarithromycin 500 mg bid or levofloxacin 500 mg qd for 7–10 days. After 6–12 weeks of triple combination therapy, repeated gastroendoscopy with rapid urease test, histology, and culture was performed. If the patient refused repeated gastroendoscopy, ^13^C-urea breath tests were conducted to assess the status of* H. pylori* infection.

This study conformed to the Helsinki Declaration and was approved by the Institutional Review Board (IRB) of Kaohsiung Medical University Hospital (IRB number: KMUH-IRB-20120327). The written informed consents were collected from all participating patients.

### 2.2. Diagnosis of* H. Pylori* Infection

#### 2.2.1. Culture Examination

Biopsy specimens were rubbed on the surface of a Columbia blood agar plate and then incubated at 35°C under microaerobic conditions for 4-5 days. Culture of* H. pylori* was considered positive if one or more colonies showed gram negativity, oxidase (+), catalase (+), urease (+), and spiral or curved rods in morphology.

#### 2.2.2. Histological Examination

The biopsy specimens were fixed with formalin, embedded in paraffin, and stained with hematoxylin and eosin. The result for the gram's stain was considered positive when curvy, gram-negative bacteria were found on the slide. They were interpreted and reported by the same pathologist.

#### 2.2.3. Rapid Urease Test

The results of the rapid urease test (CLO test, Delta West, Bentley, WA, Australia) were interpreted as positive if the color of the gel became pink or red after 6 hours at room temperature.

#### 2.2.4. ^13^C-Urea Breath Test

The ^13^C-urea was manufactured by the Institute of Nuclear Energy Research, Taiwan. One hundred milliliters of fresh whole milk was used as the test meal. This detailed procedure was reported in our previous study [10].

For patients who received endoscopy,* H. pylori* infection was established if the culture was positive or both CLO test and histology were positive.

### 2.3. The Methods of Trace Element Assay

Blood samples were drawn from the median cubital vein in individuals and collected in vacutainer plain tubes. The centrifuge was set at a speed of 3000 rpm for 15 minutes under room temperature. After centrifugation, the supernatant was pipetted into an unused vial for −20°C preservation without any additives. The serum copper, zinc, and selenium levels were determined by inductively coupled plasma mass spectrometry (ICP-MS). At present, ICP-MS is the most sensitive analytical technique for the determination of trace elements in various matrices.

### 2.4. Statistical Analysis

All data were analyzed using Statistical Package for Social Sciences Version 14.0 software (SPSS Inc., Chicago, Ill, USA). Statistical differences between mean values were tested with Student's *t-*test. The difference between the two groups was tested with paired *t-*test. A *P* value of <0.05 was considered statistically significant.

## 3. Results

There were 93 patients in our study: 63 patients infected with* H. pylori* in the study group and 30 patients without* H. pylori* infection in the control group. The mean age was 53.87 ± 13.18 years in the study group and 48.00 ± 12.22 years in the control group. The endoscopic diagnosis in the study group was 11 patients with gastric ulcers, 28 patients with duodenal ulcers, 3 patients with gastritis, and 21 patients with reflux esophagitis. The control group had 30 patients with primary endoscopic finding of gastritis (Table 1).

Table 2 shows that serum copper, zinc, and selenium levels had no significant differences between study and control groups (all *P* values > 0.05). Forty-nine patients had successful* H. pylori* eradication with a rate of 77.78% (49/63). After successful* H. pylori* eradication, the serum levels of copper, zinc, and selenium had no significant difference compared with before treatment (Table 3, all *P* values > 0.05). Among the patients with failure of* H. pylori* eradication, the serum copper and zinc levels were not significantly different between before and after treatments (Table 4, *P* = 0.26 and 0.25, resp.). However, the serum selenium level was significantly lower after failure of* H. pylori* eradication therapy (Table 4, *P* < 0.05).

## 4. Discussion

*H. pylori* infection may result in stomach inflammation.* H. pylori*, when having altered gastric secretion coupled with tissue injury, leads to peptic ulcer disease and gastritis, and maybe progresses to atrophy, intestinal metaplasia, and eventually gastric carcinoma.* H. pylori* also leads to hypochlorhydria in* H. pylori-*related gastritis [11]. The change of gastric environment may affect the absorption of trace elements.

Copper is an essential mineral in the human body, which is required as a catalytic cofactor in different enzyme reactions, such as an allosteric enzyme component and a potent antioxidant with a critical role in the oxidant defense system [12]. For children, Janjetic et al. reported that serum copper level was associated with gastric* H. pylori* infection [13]. For adults, previous reports have shown that serum copper level had no significant difference between gastric* H. pyloric* infection and noninfection [14, 15]. These results are compatible with our study, which revealed that there was no significant difference between* H. pylori-* positive and* H. pylori*-negative patients (Table 2). Even after* H. pylori* eradication therapy, the serum copper levels had no significant changes between successful and failed* H. pylori* eradication groups (Tables 3 and 4).

Zinc is an important trace element in the organism, with catalytic, structural, and regulatory roles. Zinc is also related to some diseases, including Alzheimer's disease, cancer, aging, diabetes, depression, and Wilson's disease [5]. For children, Janjetic et al. have reported that serum zinc level was not associated with gastric* H. pylori* infection [13, 16]. The role of zinc in adults seems to modulate the oxidative stress in gastric mucosa. Zinc deficiency results in an increased susceptibility to oxidative stress and higher risk of mucosal damage in inflammation [17]. It has been reported that serum zinc level was an indicator of protecting gastric mucosa against damage, and it appears significantly reduced in patients with gastritis, peptic ulcer, and gastric cancer [18]. The degree of inflammation in* H. pylori*-induced gastritis seems to be modulated by gastric tissue zinc concentration. The more severe the* H. pylori* infection is, the lower concentration of zinc in gastric mucosa is noted [19]. In our study, the serum zinc level was lower in* H. pylori*-infected patients compared with* H. pylori*-negative cases. However, the difference was not statistically significant (Table 2). Besides, our study demonstrated the* H. pylori* eradication regimen had no effect on the serum zinc levels in both successful and failed* H. pylori* eradication groups (Tables 3 and 4).

Selenium has long been noted as an integral component of glutathione peroxidase, which is an important antioxidant against oxidative damage in human body [20]. It is also involved in maintaining structure and functional efficiency of mitochondria [21]. Selenium deficiency is associated with cancer formation, immune dysfunction, cardiovascular disease, and reproductive disorder [8, 22]. Previous reports showed that serum selenium level was not associated with* H. pylori*-infected subjects [15]. However, the report of Üstündağ et al. indicated that selenium accumulated obviously in gastric tissue in the cases of* H. pylori*-related antral inflammation and then significantly decreased in tissue after successful* H. pylori* eradication [23]. Furthermore, this reactive increase in gastric mucosal selenium also disappeared in atrophic gastritis, a status of* H. pylori*-related precancerous lesion. So, the authors suggested that decreased gastric mucosal selenium during a long-lasting mucosal inflammation may be a part of the gastric microenvironmental changes in gastric carcinogenesis [23]. This is why the relationship between serum selenium level and* H. pylori*-infected subjects has been studied in this paper. It shows that serum selenium level has no significant difference between* H. pylori*-positive and* H. pylori*-negative groups (Table 2). However, the serum selenium levels decrease after* H. pylori* eradication therapy (Tables 3 and 4), especially decreasing significantly in failed* H. pylori* eradication group (Table 4, *P* < 0.05). The definite mechanism responsible for decreasing serum selenium level after* H. pylori* eradication therapy is not clearly defined. However, the changes of microbiota in small intestine may play an important role in determining serum selenium level because it is mainly absorbed in the duodenum and cecum by active transport through a sodium pump [22]. Previous report showed that short-term antibiotics use, such as* H. pylori* eradication regimen, may have a long-term impact on the native microbiota in the intestine for up to 4 years posttreatment [24]. Besides, proton pump inhibitor used in* H. pylori* eradication regimen also results in bacterial and fungal overgrowth in small intestine [25]. The changes of microenvironmental microbiota in small intestine may explain the effect of* H. pylori* eradication therapy on decreasing serum selenium level, but the detailed mechanism needs to be further elucidated.

Our study aims to explore the association of serum copper and zinc levels with* H. pylori* infection, although the relationship is not significant in statistics. However, the serum selenium level decreases after* H. pylori* eradication therapy in both successful and failed eradication groups, and it achieves significantly lower levels of selenium after eradication therapy in the failed group. Our study shows that the* H. pylori* eradication regimen may influence the serum selenium level.

## Figures and Tables

**Table 1:** The demography of studied patients including healthy control group and *Helicobacter pylori*-infected group.

	Control (*n* = 30)	Study (*n* = 63)
*Helicobacter pylori *	Negative	Positive
Age (years)	48.00 ± 12.22	53.87 ± 13.18
Male/female	16/14	36/27
Gastric ulcer	0	11
Duodenal ulcer	0	28
Gastritis	30	3
Reflux esophagitis	0	21

Control: *H. pylori*-negative patients.

Study: *H. pylori*-positive patients.

**Table 2:** The comparison of serum copper, zinc, and selenium levels between control and study groups.

	Control (*n* = 30)	Study (*n* = 63)	*P* value
Cu (mg/L)	1.11 ± 0.27	1.05 ± 0.31	0.40
Zn (mg/L)	1.92 ± 0.37	1.79 ± 0.45	0.19
Se (*μ*g/L)	142.03 ± 25.61	155.05 ± 47.77	0.17

Control: *H. pylori*-negative patients.

Study: *H. pylori*-positive patients.

**Table 3:** The comparison of serum copper, zinc, and selenium levels in *Helicobacter pylori-  (Hp-)* infected patients before and after successful eradication therapy (*n* = 49).

	Before *Hp* eradication	After *Hp* successful eradication	*P* value
Cu (mg/L)	1.01 ± 0.25	1.02 ± 0.28	0.66
Zn (mg/L)	1.78 ± 0.42	1.67 ± 0.44	0.17
Se (*μ*g/L)	154.24 ± 37.51	141.72 ± 39.01	0.06

**Table 4:** The comparison of serum copper, zinc, and selenium levels in *Helicobacter pylori-  (Hp-)* infected patients before and after failure of eradication therapy (*n* = 14).

	Before *Hp* eradication	After *Hp* failed eradication	*P* value
Cu (mg/L)	1.23 ± 0.40	1.16 ± 0.29	0.26
Zn (mg/L)	1.84 ± 0.54	1.62 ± 0.51	0.25
Se (*μ*g/L)	157.24 ± 37.51	127.20 ± 33.92	<0.05
